# Plasmacytoid Dendritic Cell Impairment in Metastatic Melanoma by Lactic Acidosis

**DOI:** 10.3390/cancers12082085

**Published:** 2020-07-28

**Authors:** Matilde Monti, Raffaella Vescovi, Francesca Consoli, Davide Farina, Daniele Moratto, Alfredo Berruti, Claudia Specchia, William Vermi

**Affiliations:** 1 Department of Molecular and Translational Medicine, University of Brescia, 25123 Brescia, Italy; m.monti002@unibs.it (M.M.); raffaella.vescovi@gmail.com (R.V.); claudia.specchia@unibs.it (C.S.); 2 Oncology Unit, ASST Spedali Civili di Brescia, 25123 Brescia, Italy; francesca.consoli@icloud.com (F.C.); alfredo.berruti@unibs.it (A.B.); 3 Radiology Unit, Department of Medical and Surgical Specialties, Radiological Sciences and Public Health, University of Brescia, 25123 Brescia, Italy; davide.farina@unibs.it; 4 Laboratory of Genetic Disorders of Childhood, Angelo Nocivelli Institute for Molecular Medicine, ASST Spedali Civili di Brescia, 25123 Brescia, Italy; daniele.moratto@gmail.com; 5 Department of Pathology and Immunology, Washington University School of Medicine, Saint Louis, MO 63101, USA

**Keywords:** plasmacytoid dendritic cells, melanoma, lactate dehydrogenase, TLR, interferon, CXCL10

## Abstract

The introduction of targeted therapies and immunotherapies has significantly improved the outcome of metastatic melanoma (MM) patients. These approaches rely on immune functions for their anti-melanoma response. Plasmacytoid dendritic cells (pDCs) exhibit anti-tumor function by production of effector molecules, type I interferons (I-IFNs), and cytokines. Tissue and blood pDCs result compromised in MM, although these findings are still partially conflicting. This study reports that blood pDCs were dramatically depleted in MM, particularly in patients with high lactate dehydrogenase (LDH) and high tumor burden; the reduced pDC frequency was associated with poor overall survival. Circulating pDCs resulted also in significant impairment in interferon alpha (IFN-α) and C-X-C motif chemokine 10 (CXCL10) production in response to toll-like receptor (TLR)-7/8 agonists; on the contrary, the response to TLR-9 agonist remained intact. In the BRAF^V600+^ subgroup, no recovery of pDC frequency could be obtained by BRAF and MEK inhibitors (BRAFi; MEKi), whereas their function was partially rescued. Mechanistically, in vitro exposure to lactic acidosis impaired both pDC viability and function. In conclusion, pDCs from MM patients were found to be severely impaired, with a potential role for lactic acidosis. Short-term responses to treatments were not associated with pDC recovery, suggesting long-lasting effects on their compartment.

## 1. Introduction

The prognosis of metastatic melanoma (MM) patients has been dramatically improved by novel therapeutic strategies including targeted therapies and immune checkpoint blockades (ICB) [1,2]. In fact, more than half of melanoma patients (about 50–60%) harbor BRAF mutation, together with the corresponding downstream signal transduction in the MAPK (mitogen-activated protein kinase) pathway [3]. Historically, the development of high selective targeted agents such as vemurafenib or dabrafenib has dramatically improved overall survival (OS), progression-free survival (PFS), and response rate in BRAF^V600+^ advanced melanoma patients, in comparison to standard chemotherapy [4,5]. Unfortunately, the great majority of patients treated with BRAF inhibitor (BRAFi) monotherapy developed secondary resistance to treatment within 6–8 months [4,5]. Blockade of CTLA-4 and PD-1 receptors expressed by lymphocytes leads to their activation against tumor cells. The anti-CTLA-4 antibody ipilimumab was the first discovered ICB, showing a plateau in the survival curve in 21% of patients [6,7]. Anti-PD-1 agents such as nivolumab and pembrolizumab improve PFS and OS in comparison to ipilimumab, with an objective response rate of about 40% [6,8]. Anti-PD-1 treatments are considered an effective option in advanced melanoma patients, regardless of BRAF mutation [9].

The MAPK pathway hyper-activation is associated with increased metastatic behavior, reduced apoptosis, and modulation of interaction between melanoma cells and the immune system [10,11]. Moreover, BRAF^V600+^ melanoma cell lines secrete immunosuppressive cytokines such as interleukin 10 (IL-10), vascular endothelial growth factor (VEGF), and interleukin 6 (IL-6), which promote the recruitment of regulatory T cells and myeloid-derived suppressor cells [12]. Mutant BRAF also downregulates the expression of melanoma differentiation antigens (MDA) and class I major histocompatibility complex (MHC-I) molecules on tumor cells, preventing their recognition by CD8^+^ T cells [13,14]. Consequently, MAPK-targeted therapy affects melanoma cell immunogenicity and immune contexture involving different effector and regulatory mechanisms [10,15]. The MAPK pathway inhibition suppresses the secretion of immunosuppressive cytokines and leads to upregulation of MDA in melanoma cell lines [12,14], improving T cell recognition and increasing intra-tumoral CD4^+^ and CD8^+^ T lymphocytes [11]. The inhibition of MAPK pathway in melanoma cells also restores cytokine secretion and co-stimulatory molecule expression by dendritic cells (DCs), rescuing their compromised function [12]. On the contrary, MEK inhibition negatively affects in vitro DC and T cell viability and function [11,16]. However, the systemic administration of BRAFi does not alter leukocyte subset frequencies [17], and the combination of BRAFi and MEKi enhances immunological activation [10]. A pre-existing immunological signature predicts the response to BRAFi/MEKi [18]. Furthermore, the MAPK activation downregulates interferon alpha receptor 1 (IFNAR1) signaling; accordingly, BRAFi reverses IFNAR1 inhibition in melanoma biopsies, providing a rationale for the combination of interferon alpha (IFN-α) with BRAFi [19].

All these findings suggest a significant role for immune cell components in mediating responses, not only to ICB, but also to targeted therapy. Among cells involved in melanoma immunity, plasmacytoid dendritic cells (pDCs) exert an important role in shaping the anti-tumor immune response. A distinctive feature of pDCs is the production of a large amount of type I interferon (I-IFN) after nucleic acid sensing through toll-like receptor (TLR) 7- and 9-dependent signaling pathways [20,21]. IFN-α production not only affects tumor cell proliferation, angiogenesis, and metastasis [22,23], but also acts on different immune cell populations involved in anticancer immunity such as NK, T, and B lymphocytes [24,25,26]. Autocrine IFN-α/β signaling also regulates the induction of interferon signature genes, among which pro-inflammatory chemokines (i.e., CXCL9, CXCL10, and CXCL11) [27], with potential antitumor activity driving TH1 polarization of immune cells [28].

By analyzing a large cohort of primary and metastatic cutaneous melanomas, we recently monitored pDC dynamic during melanoma evolution. In MM patients, pDCs become almost absent in the tumor tissues and severely reduced in their circulation, particularly in the advanced M1c group [29]. The subversion of the mechanisms leading to the systemic pDC collapse might potentiate spontaneous and drug-induced anti-melanoma immunity, providing additional benefit to the current systemic therapies. Mechanistically, exposure to melanoma cell supernatants resulted in significant death of terminally differentiated pDCs and in defective generation of pDCs from CD34^+^ progenitors [29]. This effect is dependent on soluble components released by melanoma cells, and a role by lactic acidosis microenvironment has been proposed [30,31]. The neoplastic cell metabolism shifts toward high glucose uptake and enhanced lactate production, regardless of oxygen availability, known as the Warburg effect [32]. Lactate dehydrogenase (LDH-A) is a key enzyme that converts pyruvate to lactate in the final step of the glycolytic pathway. High levels of lactate within tumor cells are exported by monocarboxylate transporters (MCT) coupled with protons exported across the plasma membrane, leading to lactic acidosis in the tumor microenvironment [33]. This leads to enhancement of tumor-associated immune-suppressive functions and inhibition of effector cells in the tumor milieu [30,31,34]. This study analyzes circulating pDCs in a prospective cohort of chemo-naïve MM patients. pDCs from MM patients are severely impaired in their frequency and function, with a potential role for lactic acidosis. Moreover, short-term responses to BRAFi and MEKi treatments are not associated with a full pDC recovery, suggesting long lasting effects on their compartment.

## 2. Results

### 2.1. Clinical Features and Outcome of the MM Cohort

Patients’ clinical characteristics at baseline (T0) are reported in Table 1 and Table S1. Patients had a median age of 60 years (range: 23–79 years) at the time of clinical diagnosis, while healthy donors (HD; *N* = 25) had a median age of 44 years (range: 25–56 years). Sixteen patients had BRAF-mutated melanomas, including BRAF p.V600E (*n* = 12) or p.V600K (*n* = 4). Seven MM were NRAS-mutated, and included p.Q61K (*n* = 2) or p.Q61R (*n* = 5). The remaining six patients were BRAF/NRAS wild-type. The median LDH level at baseline was 220 IU/L (range: 134–236 IU/L). Patients were sub-grouped into M1a (*n* = 5) + M1b (*n* = 7) and M1c (*n* = 17) categories, accordingly to the “American Joint Committee on Cancer (AJCC) Melanoma Staging and Classification 7th edition”. Sixteen patients (55.17%) were treated with targeted therapies (vemurafenib; dabrafenib; vemurafenib + cobimetinib; dabrafenib + trametinib), and 13 patients received ICB (ipilimumab or pembrolizumab) (44.83%). Complete response (CR), partial response (PR), and stable disease (SD) were achieved in 6.89%, 31.03%, and 6.89% of patients, respectively. Progression disease (PD) was observed in 55.17% of patients (43.75% in the targeted therapy group and 56.25% in the ICB group).

### 2.2. Peripheral Blood Immune Populations and pDC Function Were Impaired in Chemo-Naïve MM Patients

As previously reported, circulating pDCs are reduced in various human cancer patients compared to healthy subjects, particularly in the advanced stage of disease [35]. By flow cytometry on fresh whole blood, we previously documented the collapse of pDC and myeloid DC (mDC) subsets in advanced MM [29]. We extended this finding, showing that CD3^+^ and CD4^+^ T lymphocytes remained unchanged in MM (Table 2). We could not detect significant differences on the basis of the molecular profile of the tumor both for absolute number of total leukocytes and for the frequencies of all analyzed immune cell populations (Table S2). Altogether, these data suggest that MM patients are characterized by an impairment of blood DCs, but not T lymphocytes.

**Figure 1:**
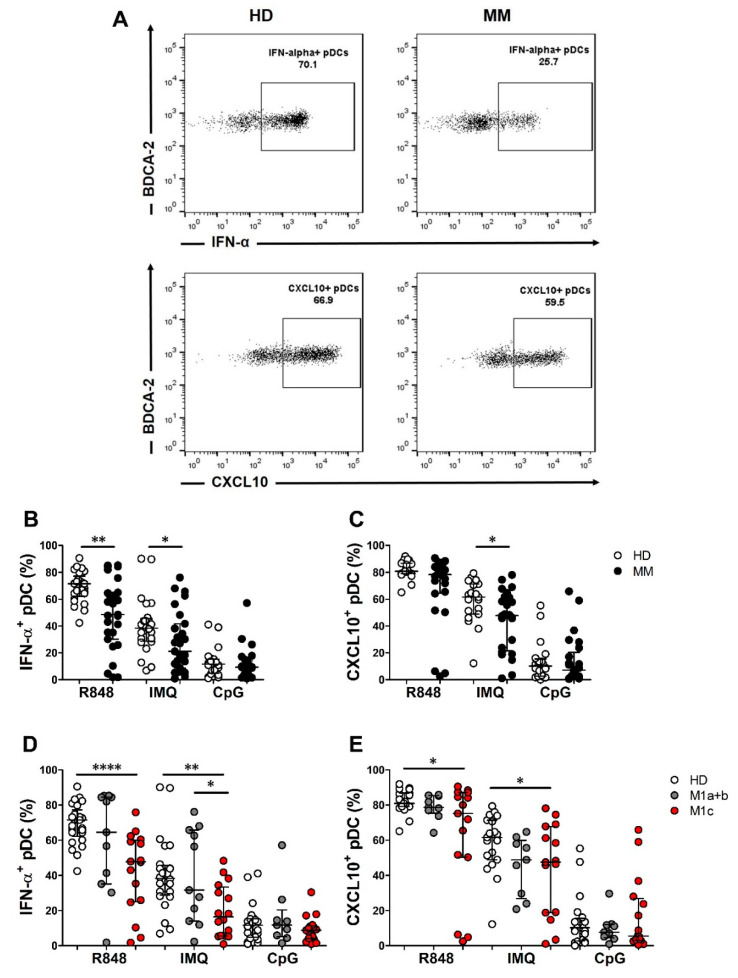
Frequency of interferon alpha (IFN-α) and CXCL10-producing plasmacytoid dendritic cells (pDCs) in chemo-naïve MM patients and HD. Representative dot plots of R848-stimulated IFN-α^+^ and CXCL10^+^ pDC subsets obtained from HD and MM patients are shown (**A**). PBMCs were isolated from peripheral blood of HD (*n* = 25) and MM patients (*n* = 29). Total PBMCs were cultured in RPMI 1640 medium supplemented with 10% FBS and IL-3 and stimulated with R848 or IMQ for 2 h (**B**,**D**) and 6 h (**C**,**E**), and with CpG-ODN 2216 for 6 h (**B**–**E**). IFN-α (**B**,**D**) and CXCL10 (**C**,**E**) were analyzed by intracellular flow cytometry staining. Scatter dot plot graphs illustrate the percentages of positive pDCs evaluated on BDCA-2^+^/CD123^+^ cells. Subgroup analysis of the MM cohort illustrating the frequency of IFN-α^+^ and CXCL10^+^ pDCs in M1a-c categories (**D**,**E**). Median and IQR are shown in (**B**,**C**). Mean and SD are shown in (**D**,**E**). The statistical significance was calculated by Wilcoxon–Mann–Whitney test (**B**,**C**) and by a Student’s *t*-test (**D**,**E**). * *p* < 0.05; ** *p* < 0.01; **** *p* < 0.0001.

We subsequently tested the response of pDCs to TLR-7/9 agonist stimulation. I-IFN exerts cancer cell intrinsic effects and modulates the immunoediting process [36,37,38], while CXCL10, also known as interferon gamma inducible protein 10 (IP-10), is a pro-inflammatory chemokine that is relevant for the recruitment of antigen-specific T cells into the tumor tissues [39]. Through using intracellular flow cytometry, we analyzed the pDC proficiency to produce IFN-α and CXCL10, after in vitro stimulation of peripheral blood mononuclear cells (PBMCs) with TLR-7/9 agonists (Figure 1A). On a background of patients’ heterogeneity, stimulation with two different TLR-7 agonists resulted in a significant reduction of the frequency of IFN-α-producing pDCs in MM (R848 *p* = 0.004; imiquimod (IMQ) *p* = 0.02) (Table 2 and Figure 1B). Moreover, the percentage of CXCL10 producing pDCs was also significantly reduced after IMQ exposure (*p* = 0.03) (Table 2 and Figure 1C). On the contrary, under CpG-A oligodeoxynucleotides (ODN) stimulation, IFN-α and CXCL10 production were not significantly different between MM and HD (Table 2 and Figure 1B,C). The defect in TLR-7 signaling response was not correlated with the melanoma molecular profile in the cohort analyzed herein (Table S2).

According to the melanoma staging system (seventh edition, AJCC), our cohort of MM patients was classified into M1a+b and M1c categories (Table S1) [40]. Compared to HD and M1a+b, the M1c subgroup showed a significant decrease in IFN-α-producing pDCs after TLR-7 stimulation (R848: HD vs. M1c, *p* < 0.0001; IMQ: HD vs. M1c, *p* = 0.0014; M1a+b vs. M1c, *p* = 0.0190) (Figure 1D). Similarly, the percentage of CXCL10-producing pDCs resulted in a significant decrease in advanced disease stages (R848: HD vs. M1c, *p* = 0.0133; IMQ: HD vs. M1c, *p* = 0.0485) after TLR-7 agonist administration (Figure 1E). Altogether, these data highlight an impairment of the TLR-7 signaling pathway in fully differentiated pDCs that are associated with disease progression.

### 2.3. LDH Level and Tumor Burden Were Associated with Decreased Frequency of Peripheral Blood Immune Cells in Chemo-Naïve MM Patients

**Figure 2:**
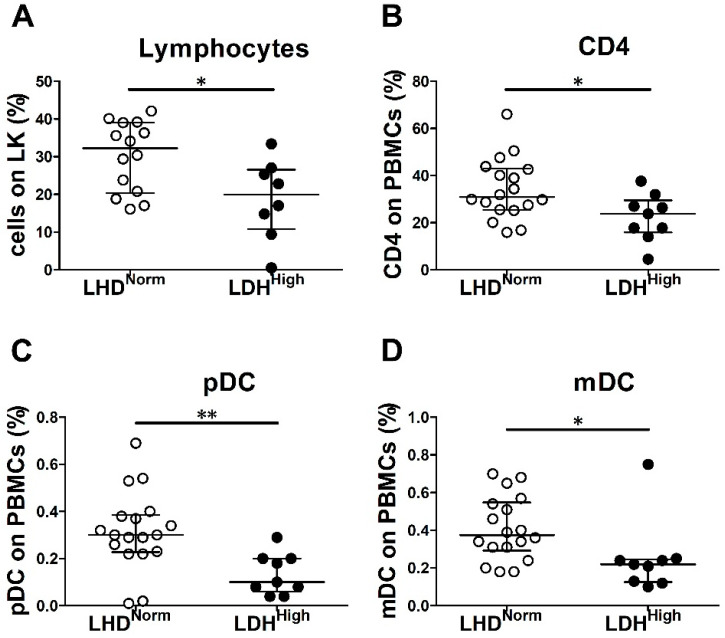
High LDH serum level associated with decreased frequencies of peripheral blood immune cells in MM patients. Immune cell populations were identified on whole blood samples of chemo-naïve MM patients by cell counts or flow cytometry. Scatter dot plot graphs represent the percentages of lymphocytes on total leukocytes (LK) (**A**), CD4^+^ T lymphocytes (**B**), pDCs (**C**), and mDCs (**D**) on total PBMCs. LDH levels are indicated as LDH^Norm^ (for values within the normal range) and LDH^High^ (for values greater than the normal range). Error bars represent the median with IQR (*n* = 27). The statistical significance was calculated by Wilcoxon–Mann–Whitney test. * *p* < 0.05; ** *p* < 0.01.

Elevated level of serum LDH is a relevant independent negative prognostic biomarker in melanoma that indicates an active metastatic disease [41]. The analysis of immune cells in the peripheral blood of chemo-naïve patients revealed that levels of LDH above the normal range (details are found in the Materials and Methods section) are associated with a significant decreased frequency of circulating lymphocytes (*p* = 0.02), particularly of CD4^+^ T cells (*p* = 0.03) (Table 3 and Figure 2A,B). Similarly, a decline in the frequencies of pDCs and mDCs resulted in association with elevated serum levels of the enzyme (*p* = 0.003 and *p* = 0.02, respectively) (Table 3, Figure 2C,D). These data suggest that the systemic release of LDH in advanced melanoma might affect the generation or viability of blood T lymphocytes, pDCs, and mDCs. On the contrary, serum LDH levels are not associated with a reduced frequency of IFN-α^+^ and CXCL10^+^ pDCs in response to TLR-7/9 agonists in chemo-naïve MM patients (Table 3).

Tumor burden at baseline has been identified as a relevant predictor for MM outcome [42]. Soluble products or metabolic competition by massive amounts of melanoma cells could explain the defective immune cell frequencies and function. Accordingly, tumor burden resulted in inverse correlated with the frequencies of circulating lymphocytes (*p* = 0.03), including CD3^+^ and CD4^+^ T cells (*p* = 0.04 and *p* = 0.02, respectively), as well as pDC and mDC frequencies (*p* = 0.006 and *p* = 0.001, respectively) (Table 4). On the contrary, no correlation was detected between the tumor burden and the pDC function (Table 4).

In conclusion, systemic LDH and bulky disease are associated with impaired immune cell generation and viability, particularly in DCs.

### 2.4. In Vitro Exposure to Lactic Acidosis Impaired the Viability and Function of Fully Differentiated pDCs

**Figure 3:**
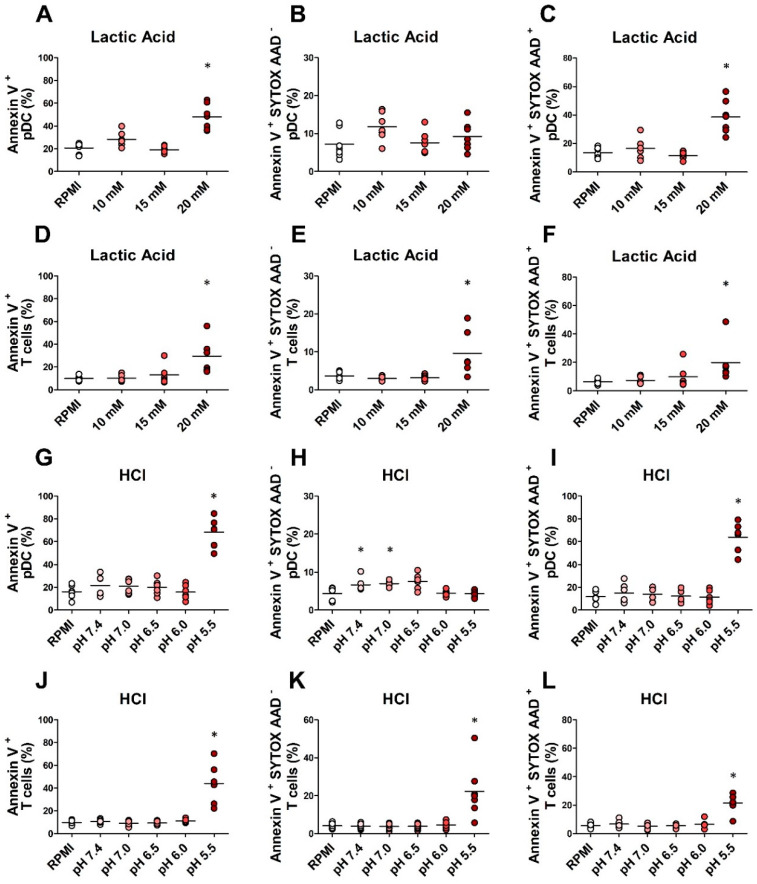
Lactic acidosis affects the viability of pDCs and T cells. pDCs and T cells purified from buffy coats of HD were cultured in RPMI 1640 medium supplemented with 10% FBS plus lactic Acid (10 mM; 15 mM; 20 mM) (*n* = 7 (**A**–**C**); *n* = 6, (**D**–**F**)) or hydrochloric acid (pH = 7.4; 7.0; 6.5; 6.0; 5.5 (*n* = 6 (**G**–**I**); *n* = 7, (**J**–**L**)) for 24 h. IL-3 was added to pDCs’ culture. The cellular viability was analyzed by annexin V/SYTOX AADvanced staining in flow cytometry. Aligned dot plot graphs show the percentages of dead (**A**,**D**,**G**,**J**), early apoptotic (**B**,**E**,**H**,**K**), and late apoptotic or necrotic cells (**C**,**F**,**I**,**L**). Bars represent the mean of biological replicates. The statistical significance was calculated by two-sample paired sign test. * *p* < 0.05.

The oncometabolite lactate and acidosis have immunosuppressive effects on various immune cells, including pDCs [43,44,45]. Lactate, glucose concentrations, and the pH level were measured in the supernatants of human melanoma cell lines (SN-mel; collected as previously described by Vescovi et al. [29]). Compared to RPMI control, SN-mel contained higher levels of lactate, inversely associated with glucose levels (Figure S1A), and slight acidosis (pH range: 6.7–7.2 versus 8.0) (Figure S1B), indicating metabolic lactic acidosis. We tested the viability of pDCs and T lymphocytes in three different culture conditions: (i) lactic acidosis, (ii) lactosis, and (iii) acidosis. To this end, we exposed pDCs and T lymphocytes purified from HD to increasing concentrations of lactic acid (LA), sodium lactate (NaL), and hydrochloric acid (HCl). The pH values of the medium containing LA 10 mM, 15 mM, and 20 mM corresponded to 6.5, 6.0, and 5.5, respectively. pDC death, in the form of late apoptosis or necrosis, was significantly increased by high concentrations of LA (20 mM; *p* = 0.01) (Figure 3A–C). Similarly, the viability of T cells was significantly reduced by the highest lactic acidosis condition (*p* = 0.03), in terms of both apoptotic (*p* = 0.03) and necrotic cell death (*p* = 0.03) (Figure 3D–F). The highest concentration of HCl (pH = 5.5) was also associated with a significant increase of the pDC late apoptosis or necrosis (*p* = 0.03), while affecting both late (*p* = 0.01) and early apoptosis (*p* = 0.01) of T cells (Figure 3G–L). On the contrary, the lactosis condition did not affect pDC and T cell viability (Figure S2). Together these results indicate that acidosis, but not lactosis, induces pDC and T cell death.

**Figure 4:**
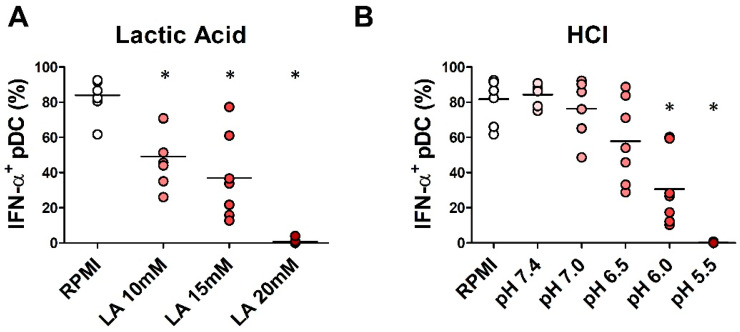
In vitro lactic acidosis affects IFN-α production by pDCs. pDCs purified from buffy coats of HD were cultured in RPMI 1640 medium supplemented with 10% FBS and IL-3 plus lactic acid (10 mM; 15 mM; 20 mM) (*n* = 7; (**A**)) or hydrochloric acid (pH = 7.4; 7.0; 6.5; 6.0; 5.5) (*n* = 7; (**B**)) for 24 h. pDCs were stimulated with R848 for 2 h. Intracellular IFN-α was analyzed by flow cytometry. Aligned dot plot graphs show the percentages of IFN-α^+^ pDCs evaluated on BDCA-2^+^/CD123^+^ cells. Bars represent the mean of biological replicates. The statistical significance was calculated by two-sample paired sign test. * *p* < 0.05.

Lactate has been recently proposed as an inhibitor of the pDC function in the tumor microenvironment [45,46]. Hence, we assessed the IFN-α production on pDCs isolated from HD and exposed to increasing concentrations of LA. The percentage of IFN-α-producing pDCs under R848 stimulation was progressively reduced by increasing concentrations of LA (10 mM, *p* = 0.01; 15 mM, *p* = 0.01; 20 mM, *p* = 0.01) and was completely abolished at a concentration of 20 mM (Figure 4A). pDCs were subsequently exposed to increasing concentration of HCl and stimulated with R848. The percentage of IFN-α^+^ pDCs was progressively reduced by decreasing pH levels as well (pH 6.0, *p* = 0.01; pH 5.5, *p* = 0.01) (Figure 4B). In conclusion, in vitro acidosis dramatically impaired the pDC viability and their proficiency to produce IFN-α.

### 2.5. Baseline Lymphocyte and pDC Frequencies Predicted MM Outcome

**Figure 5:**
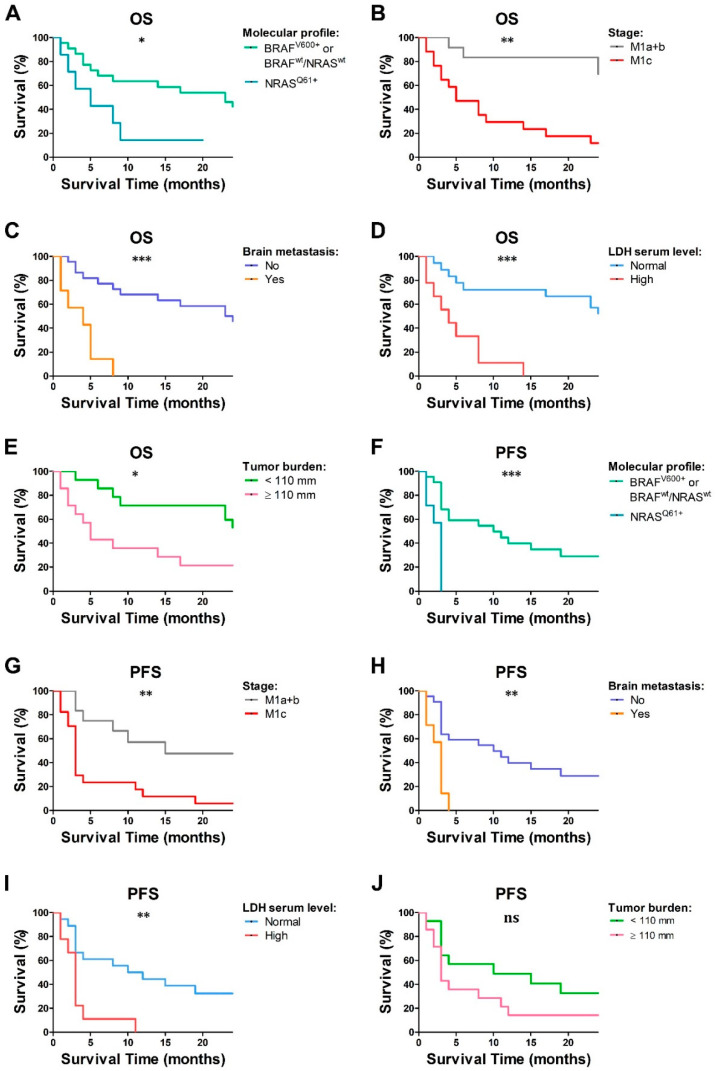
Survival analysis of MM patients. Overall survival (OS) (**A**–**E**) and progression-free survival (PFS) (**F**–**J**) according to molecular profile (*N* = 29; (**A**,**F**)), stage of melanoma (*N* = 29; (**B**,**G**)), presence of brain metastases (*N* = 29; (**C**,**H**)), LDH serum level (*N* = 27; (**D**,**I**)), and tumor burden (*N* = 28; (**E**,**J**)) are reported. Survival analysis was performed using the Kaplan–Meier method and the statistical significance was calculated by the log-rank test. * *p* < 0.05; ** *p* < 0.01; *** *p* < 0.001; ns = not statistically significant *p*-value.

**Figure 6:**
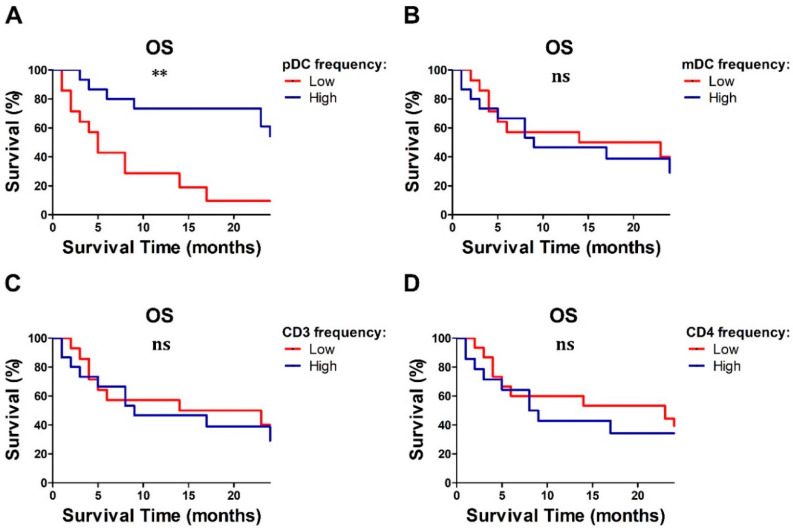
Survival analysis of MM patients according to immune cell subsets. Overall survival analysis (OS) according to the frequency of pDCs (**A**), mDCs (**B**), CD3^+^ (**C**), and CD4^+^ T lymphocytes (**D**) (*N* = 29) are reported. Survival analysis was performed using the Kaplan–Meier method and the statistical significance was calculated by the log-rank test. ** *p* < 0.01; ns = not statistically significant *p*-value.

On the basis of the clinical relevance of recent treatment options for advanced stage of disease, additional prognosticators might help in patient’s selection. The analysis of survival revealed that among 29 MM patients, the median OS time was 14 months (range: 1–35 months), with a total of 62% of patients dying, while the median PFS time was 4 months (range: 1–35 months) (Table S3). Among the significant prognosticators, NRAS mutations (*p* = 0.03), M1c stage (*p* = 0.0016), presence of brain metastases (*p* = 0.0001), high LDH levels (*p* = 0.0002), and elevated tumor burden (*p* = 0.01) resulted in a significant correlation with poor outcome in univariate analysis (Figure 5A–E). In terms of immune profile, a reduced frequency of lymphocytes (*p* = 0.01), pDCs (*p* = 0.01), CD3^+^ (*p* = 0.03), and CD4^+^ T cells (*p* = 0.01) predict poor OS in an univariate Cox regression model (Table 5). By subgrouping the MM cohort on the basis of the frequencies of immune cell subsets, only the low frequency of pDCs resulted in an association with worse prognosis (*p* = 0.03; Figure 6). Finally, a decrease of IFN-α and CXCL10-positive pDCs after stimulation with R848 (*p* = 0.04 and *p* = 0.03, respectively) also predicted a worse outcome. In our cohort, the PFS probability calculated in an univariate Cox regression model was significantly correlated with patient’s molecular profile (*p* = 0.0006), stage (*p* = 0.004), brain metastasis (*p* = 0.001), and LDH serum level (*p* = 0.003) (Figure 5F–J); on the contrary, the immune profile failed to predict PFS (Table S4).

These data suggest that profiling circulating immune cells, particularly pDCs, might offer additional biomarkers of outcome.

### 2.6. Partial pDC Recovery after BRAFi and MEKi Administration

**Figure 7:**
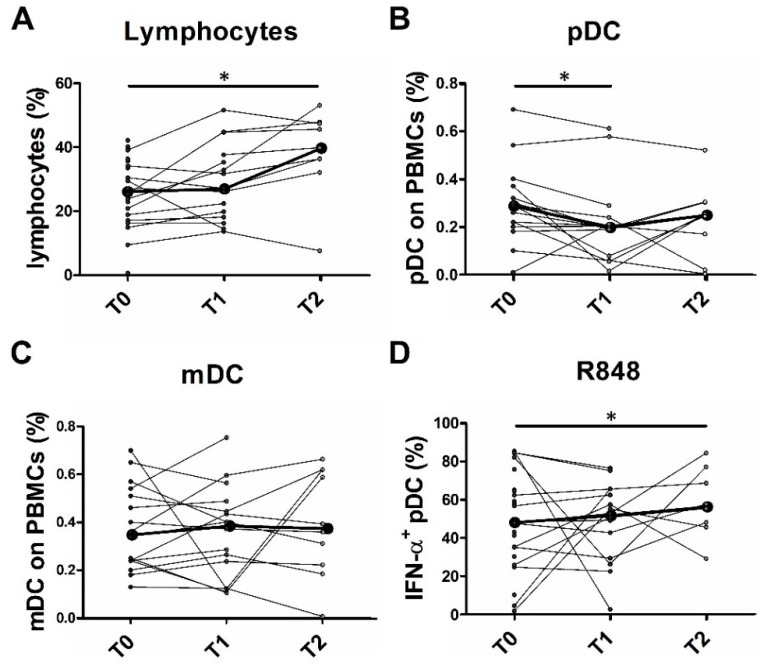
Frequency of peripheral blood immune cells and IFN-α^+^ pDCs in BRAF^V600+^ MM patients over therapy administration. Cell counts (**A**) and flow cytometry (**B**,**C**) analysis were performed on whole blood from BRAF^V600+^ MM patients before therapy initiation (T0; *n* = 16), after 30 days (T1; *n* = 12), and after 120 days (T2; *n* = 9) from therapy administration (**A**–**C**). Total PBMCs isolated from peripheral blood of MM patients were cultured in RPMI 1640 medium and stimulated with R848 for 2 h (**D**). IFN-α was analyzed by intracellular flow cytometry staining (**D**). Before–after graphs illustrate the frequency of lymphocytes (**A**), pDCs (**B**), and mDCs (**C**) on total PBMCs, and the frequency of IFN-α^+^ pDCs on BDCA-2^+^/CD123^+^ cells (**D**) for each subject. Bold black lines represent the median values. The statistical significance was calculated by Wilcoxon signed-rank test. * *p* < 0.05.

A combination of BRAFi and MEKi represents the standard of care for BRAF^V600+^ MM patients. These agents inhibit the constitutively activated RAF–RAS–MEK–ERK pathway in melanoma cells [47] and are associated with potent clinical responses [48,49], partially mediated by the immune system [10,15], including DCs [16]. We monitored peripheral blood leukocyte frequencies and pDC function in BRAF^V600+^ MM patients treated with a combination of MEKi and BRAFi or BRAFi alone. For this purpose, blood samples were obtained at different time points over treatment, on the basis of the expected time to the clinical response (details in the Materials and Methods Section 4.1). Compared to the values found in HD, no recovery in the frequencies of peripheral blood immune populations was observed. However, within the MM cohort, some differences emerged by comparing various time points. A significant increase of circulating lymphocytes was observed over 120 days of treatment (*p* = 0.05) (Table 6 and Figure 7A). Instead, pDC frequency was significantly reduced after 30 days of therapy (*p* = 0.01) but returned to the baseline level (T0) after 120 days of treatment (Table 6 and Figure 7B). No significant variation in cell frequency was detected for the other immune populations (Table 6 and Figure 7C).

Recent studies suggest that the MAPK pathway inhibition by targeted therapies in BRAF^V600+^ melanoma patients can improve the melanoma-specific immune responses [15]. In monocyte-derived DCs (moDC), interleukin 12 (IL-12) and tumor necrosis factor alpha (TNF-α) production and co-stimulatory molecule expression is impaired after co-culture with melanoma cells, but it can be easily restored by pre-treatment with BRAFi [16]. Moreover, inhibitors of MEK1/2 (i.e., PD0325901 and U0126) significantly increase the TLR-9-mediated production of I-IFN in pDCs, restoring the I-IFN production previously blocked via B cell receptor (BCR)-like signaling [50]. We examined the function of pDCs from MM patients treated with BRAFi alone or in combination with MEKi. A significant increase in the percentage of IFN-α^+^ pDCs was registered, comparing T2 versus T0 patients (*p* = 0.03) after R848 stimulation (Table 6 and Figure 7D). In contrast, no differences were detected in the pool of IFN-α-producing pDCs after IMQ or CpG stimulation. No recovery of the percentage of CXCL10-producing pDCs following TLR-7 and TLR-9 stimulation was obtained after 1 month and 4 months of therapy administration (Table 6). Taken together, these data suggest a partial rescue of the pDC function, in terms of the percentage of IFN-α producing pDCs after R848 stimulation. The inhibitory effects of MEKi on T cells and moDC cytokine production, co-stimulatory molecule expression, and viability have been previously demonstrated [10,51]; on the contrary, no effects have been reported by direct exposure of immune cells to BRAFi. No data are available on the pDC compartment. In light of these reports, we exposed fully differentiated pDCs from HD to the BRAFi PLX4032 (vemurafenib) treatment alone or in combination with the MEKi U0126. The percentage of dead pDCs, as measured by annexin V/SYTOX AADvanced staining, did not significantly increase after 24 h of treatment with PLX4032 with or without U0126 compared to the vehicle control (Figure S3A–C). Furthermore, after 24 h of BRAFi and MEKi treatment, purified pDCs were stimulated with R848, IMQ, and CpG and the IFN-α and CXCL10 intracellular production was evaluated by flow cytometry analysis. Although variable under R848 and IMQ stimuli, the percentage of IFN-α^+^ pDCs was not significantly affected by direct exposure to PLX4032 and U0126 compared to vehicle control (Figure S3D). Similarly, the percentage of CXCL10^+^ pDCs was unchanged after PLX4032 in vitro administration both alone or combined with U0126 (Figure S3E).

Altogether, these results rule out inhibitory effects of direct exposure of BRAFi and MEKi on the pDC recovery in patients undergoing systemic cancer treatment.

## 3. Discussion

The frequency of circulating pDCs is dramatically reduced in melanoma patients with systemic spread [29,52,53], but their clinical significance as well as their functional state have been poorly characterized [35]. Findings from retrospective analysis are conflicting [35]. This study reports the analysis of the circulating pDCs in a prospective chemo-naïve MM patient cohort. Results indicate that pDCs are dramatically depleted in MM patients with elevated serum LDH and high tumor burden. By comparison with HD, we found that the residual pDCs result in severe impairment in IFN-α and CXCL10 production in response to TLR-7/8 agonists, particularly in more advanced disease stage. On the contrary, the ability to respond to TLR-9 agonists remained intact. By multiple time point monitoring, we also found that in the BRAF^V600+^ subgroup, the pDC frequency was not recovered by BRAFi/MEKi treatment, whereas pDC function was partially restored. Finally, in vitro exposure to lactic acidosis negatively affected both the viability and function of terminally differentiated pDCs. One limitation of this study was the small sample size of the study cohort; however, the obtained findings provide the rationale for a prospective large-scale study.

The role of pDCs in cancer immunity is relevant due to their capability to produce large amounts of I- and III-IFNs, when properly activated through TLR-7 and -9 agonists, linking the innate and adaptive immune responses [54]. However, tumor-conditioned pDCs contribute to the establishment of an immunosuppressive milieu in several types of cancer and are associated with poor outcome [55,56,57]. IFNs participate in the host anti-tumor immune responses by exerting various regulatory functions on tumor cells as well as on cells of the microenvironment, especially on immune cells [36,58]. Here, we found that circulating pDCs are severely impaired in IFN-α and CXCL10 production in MM patients, suggesting that tumor-mediated I-IFN inhibitory mechanisms might take place in the blood. Even though the pDC response is largely heterogeneous in human subjects, we found a reduced fraction of IFN-α-producing pDCs after stimulation with TLR-7 agonists (i.e., resiquimod and imiquimod), in chemo-naïve MM patients. On the contrary, pDCs remained proficient after stimulation with a TLR-9 agonist (i.e., CpG-A ODN 2216). From a mechanistic point of view and as extension to the current study, we envisage the analysis of TLR-7 and TLR-9 protein expression on peripheral blood pDCs from MM patients. Despite the structural and functional similarities between TLR-7 and TLR-9, recent findings suggest that they are distinctly regulated in intracellular localization and trafficking by the molecular chaperone UNC93B1 [59,60]. Moreover, a contaminating pre-DC subpopulation, unable to produce high amounts of I-IFN in response to TLR7/8 and TLR9 stimulation, has been recently documented within the pDC fraction [61]. Although CpG-ODN is considered the most efficient stimulus for the TLR-9 signaling pathway activation [62], only a limited fraction of CpG-activated pDCs in HD showed an intracellular positivity for IFN-α. Human blood pDCs diversify into functionally distinct subsets after activation by CpG-ODN; the production of IFN-α by individually stimulated pDCs is controlled by stochastic gene regulation and paracrine I-IFN signaling in the microenvironment and might play a protective role reducing I-IFN levels and tissue damage [63,64].

It is worth noting that the M1c subgroup of MM was characterized by a more severe impairment of TLR-7-activated pDCs in the IFN-α and CXCL10 production, indicating a progressive reduction of the pDC function associated with advanced disease stages. Moreover, the functional impairment of the R848-activated pDCs predicted a worse prognosis in term of OS. On the other hand, neither the high LDH serum level nor the elevated tumor burden directly affected the pDC capability to produce IFN-α and CXCL10, recognizing their poor values as reliable biomarkers for pDC dysfunction in melanoma patients. On the basis of these results, assays measuring pDC function should be incorporated in future analysis to confirm their clinical relevance and utility.

Lactate and acidosis, generated by high glycolytic tumor metabolism, lead to a local suppressive effect on various immune cells, including macrophages, myeloid derived suppressor cells, and DCs [30,34,65]. The oncometabolite lactate has been recently proposed as a suppressor of IFN-α production, capable of reprogramming intra-tumoral pDCs to tolerogenic function [45,46]. High serum LDH predicts poor survival [66] and poor clinical response to anti-PD-1 treatment in melanoma patients [67], suggesting effects on cancer cell elimination by adaptive immunity [31]. A collapse of the circulating DC compartments has been previously demonstrated in advanced melanomas, more dramatically in the systemic disease (M1c) [29,52,53]. In the MM cohort analyzed in this study, the low frequencies of pDC, mDC, and T lymphocyte subsets were also associated with high serum LDH and elevated tumor burden as predictors of shorter OS. Accordingly, in vitro studies presented here hint that the exposure of pDCs and of T cells to severe acidosis promote their cell death. At the same time, increasing concentration of lactic acid as well as HCl have progressively reduced IFN-α production by fully differentiated pDCs. We found that increased lactate production was associated with a reduction of the glucose concentration in the melanoma supernatants, suggesting an active glycolytic activity by melanoma cells and nutrient deprivation in cell culture medium. On the contrary, only a slight acidosis was measured on SN-mel. Altogether, these findings support the hypothesis that an increased lactic acidosis induced by high glycolytic tumor metabolism is involved in the melanoma-mediated pDC collapse. On the other hand, as previously reported, glucose deprivation does not interfere with viability of human pDCs, whereas glycolysis is an essential metabolic pathway for pDCs to execute innate immune functions (e.g., IFN-α secretion) [68,69], suggesting that nutrient deprivation or glycolysis inhibition could impair pDC function in MM. Like many other cancer types, rapidly proliferating melanoma cells utilize aerobic glycolysis at high rates. Aerobic glycolysis, the resulting lactate and proton secretion in the tumor microenvironment as well as the levels of MCT1 transporter in melanoma cells cooperate to promote metastatization in several ways [70,71], as well as to suppress the immune surveillance. The I-IFN deficiency of pDCs might likely result from defective TLR-7/9 signaling pathways induced by soluble factors released in high amounts in melanoma patients, such as cytokines [72,73]. In addition, the I-IFN response is fine-tuned by a set of surface interferon inhibitory receptors, such as the immunoglobulin-like transcript 7 (ILT-7) and the type II C-type lectin BDCA-2 [74,75], and their physiological ligands have been identified on tumor cells [76,77]. Tumor-associated pDCs can be reprogrammed to their anti-tumor function upon appropriate re-activation with TLR-7 and TLR-9 agonists [78,79,80,81], and numerous clinical trials (phase I-III) are ongoing (Clinicaltrials.gov study identifiers: NCT02644967, NCT03445533, NCT03052205, NCT03084640, NCT03618641, NCT02680184, NCT03831295, and NCT02521870). Our study could help to identify a proper window for clinical intervention of these compounds in combination with the current therapies in MM patients. In particular, our data suggest a defective signaling through TLR-7, but not TLR-9. On the basis of these findings, administration of synthetic ODNs might represent a valid alternative to topical administration of IMQ.

The molecular profile of cancer cells can modify cancer cell immunogenicity [82] and the surrounding immune contexture [11,83]. The occurrence of BRAF^V600+^ mutation is associated with an increased pDC density in melanoma metastasis compared with BRAF wild-type tumors [84], whereas in locally advanced PCM, a collapse of the pDC compartment particularly occurs in NRAS-mutated tumors [29]. In this study, the frequencies of peripheral blood DCs or T lymphocytes were not associated with the tumor molecular profile, as was the impaired production of IFN-α by pDCs. The analysis was extended to BRAF^V600+^ MM patients treated with dabrafenib plus trametinib, vemurafenib plus cobimetinib, or vemurafenib/dabrafenib alone, which act on the constitutively activated RAF–RAS–MEK–ERK pathway [47]. Targeted therapies represent the treatment of choice in patients with bulky and symptomatic disease who deserve a rapid clinical response [9]. The therapeutic activity of BRAFi and MEKi partially depends on host immune cell activation. Indeed, BRAFi post-treatment biopsies are characterized by an increased T cell infiltration [15], and immune checkpoint molecules are increasingly expressed by tumor cells and T cells within 2 weeks of therapy [10]. Moreover, in vitro study has demonstrated that combination of vemurafenib and MEKi U0126 promotes the recovery of DC functions impaired by melanoma cells [16]. Although an objective clinical response by RECIST was observed in many BRAF^V600+^ MM patients undergoing targeted therapy, no circulating pDC and mDC recovery was noticed after 120 days of treatment by time point analysis, while a slight lymphocyte restoration was achieved. It would be relevant to test long-term responder for a better understanding of the mechanisms sustaining DC recovery. On the contrary, a moderate increase of the frequency of IFN-α-producing pDCs was obtained in patients after 4 months from therapy initiation, although the pDC function was not fully recovered. The lack of DC recovery in BRAF^V600+^ patients might be explained by some direct effects of MEKi on DC compartment, as previously demonstrated [16]. However, in vitro experiments from this study showed that pDC viability and function were not significantly affected by direct exposure to BRAFi and MEKi. Microscopic residual disease might interfere with a full pDC recovery; this suggests that combined circulating tumor DNA (ctDNA) and DC monitoring in the setting of a prospective analysis might provide support to this notion.

## 4. Materials and Methods

### 4.1. Experimental Design

This study included a prospective cohort (BRAF-mutated (*n* = 16) and BRAF/NRAS wild-type patients (*n* = 13)) for a total of 29 histologically confirmed metastatic melanoma (MM) patients (AJCC, Stage IV, Chicago, IL, USA) and 25 healthy donors (HD), enrolled between December 2014 and November 2017. The local ethics committee provided formal approval to this project (WV-Immunocancer 2014 to W.V., institutional review board code NP906). Exclusion criteria included immune deficiency (steroid administration); bone marrow transplant; and known history of human immunodeficiency virus 1 and 2 (HIV1; HIV2), hepatitis B virus (HBV), and hepatitis C virus (HCV) infection.

Patients with BRAF p.V600E/K mutant MM were monitored at different time points (T0 = 0 day, *n* = 16; T1 = 30 days, *n* = 12; T2 = 120 days, *n* = 9). HD were enrolled at the same time as chemo-naïve MM patients. A primary stratification according to the “AJCC Melanoma Staging and Classification 7th edition” was applied to our MM cohort (Table S1). Furthermore, patients were stratified on the basis of baseline LDH: values below or equal to 1 × the upper limit of normal (ULN) were identified as “normal” (*n* = 18), and values above 1 × ULN were identified as “high” (*n* = 9) (Table S1). The “tumor burden” of 28 patients who had at least 10 measurable lesions was analyzed on computed tomography scans of the chest and abdomen (Table S1). Lung nodules ≤ 5 mm in axial diameter were excluded. Baseline disease burden was determined by the sum of the product of axial diameter for the biggest metastatic lesions. The objective response was defined as complete response (CR), partial response (PR), stable disease (SD), or progressive disease (PD), according to RECIST 1.1 criteria (Table S1).

### 4.2. Human Subjects and Blood Specimen Processing

A total of 10 mL of whole blood was collected from 29 MM patients and 25 HDs. Blood was drawn directly into S-Monovette 2.7 mL K3E (1.6 mg EDTA/mL; Sarstedt, Nümbrecht, Germany), gently rocked at room temperature until processing. Additionally, the blood counts were performed on MM samples as part of their clinical routine hematology. Clinical features of the MM patients previously reported by Vescovi R. et al. [29] are included as Table S1.

### 4.3. Peripheral Blood Mononuclear Cell Stimulation

Peripheral blood mononuclear cells (PBMCs) were obtained from HD and MM patients by Ficoll gradient. PBMCs (1 × 10^6^ cells/mL) were cultured in RPMI 1640 medium (Biochrom GmbH, Berlin, Germany) with 10% fetal bovine serum (FBS) (Biochrom GmbH, Holliston, MA, USA) and 20 ng/mL human IL-3 (Miltenyi Biotec, Bergisch Gladbach, Germany). Total PBMCs (1 × 10^6^ cells/mL) were stimulated with resiquimod (R848) or imiquimod (IMQ) 5 μg/mL (Invivogen, San Diego, CA, USA) and CpG-ODN 2216 6 μg/mL (Miltenyi Biotec, Bergisch Gladbach, Germany) for 2 h and 6 h, respectively. Brefeldin A (1 μg/mL; Sigma-Aldrich, St. Louis, MO, USA) was added after 1 h or 4 h (in samples stimulated for 2 h or 6 h, respectively).

### 4.4. Purification, Culture, and Stimulation of Peripheral Blood pDCs and T Lymphocytes

PBMCs were obtained from buffy coats of healthy volunteer blood donors (courtesy of the Centro Trasfusionale, ASST Spedali Civili, Brescia, Italy) by Ficoll gradient. Peripheral blood pDCs and T cells were magnetically sorted with the Plasmacytoid Dendritic Cell Isolation Kit II and the human Pan T Cell Isolation Kit (Miltenyi Biotec, Bergisch Gladbach, Germany), respectively. Isolated pDCs and T lymphocytes (5 × 10^5^ cells/mL) were cultured in RPMI 1640 medium (Biochrom GmbH, Holliston, MA, USA) with 10% FBS (Biochrom GmbH, Holliston, MA, USA), and 20 ng/mL human IL-3 (Miltenyi Biotec, Bergisch Gladbach, Germany) was added to pDCs’ culture medium.

pDCs and T lymphocytes were cultured for 24 h in RPMI 1640 medium supplemented with 10% FBS (Biochrom GmbH, Holliston, MA, USA), containing lactic acid (LA) (Sigma-Aldrich, St. Louis, MO, USA) or sodium lactate (NaL) (Alfa Aesar, Haverhill, MA, USA) at the concentrations of 10 mM, 15 mM, and 20 mM. The pH levels of the media were measured. In addition, pDCs and T lymphocytes were cultured in RPMI 1640 medium supplemented with 10% FBS (Biochrom GmbH, Holliston, MA, USA), titrated to pH ≈ 7.4, 7.0, 6.5, 6.0, and 5.5 using HCl. A total of 20 ng/mL human IL-3 (Miltenyi Biotec, Bergisch Gladbach, Germany) was added to the pDC culture medium.

pDCs were treated with 1 μM of the BRAF Inhibitor PLX4032 (Selleck Biochem, Houston, TX), with or without 12.5 μM of the MEK inhibitor U0126 (Merck Millipore, Darmstadt, Germany), and the 0.2% of DMSO in RPMI 1640 medium was used as vehicle control.

pDCs were stimulated with 5 μg/mL of R848 or IMQ (Invivogen) and 6 μg/mL of CpG-ODN 2216 (Miltenyi-Biotec) for 2 h and 6 h. Brefeldin A (1 μg/mL, Sigma-Aldrich, St. Louis, MO, USA) was added after 1 h and 4 h, respectively.

### 4.5. Flow-Cytometric Analysis

Fluorescence minus one (FMO) was used to set the marker for positive cells. A baseline fluorescence control was used as a reference to set the fluorescence thresholds for positivity. The results were expressed as the percentage of positive cells. For measurement of the spectral overlaps, the fluorescence detected on all measurement channels was evaluated for single-labelled “compensation control” samples prior to the performed flow cytometryanalysis.

For whole blood staining, 200 μL of whole blood was incubated with a panel of fluorochrome-conjugated antibodies (panel #1 reported in Table S5) for 15 min in the dark at 4 °C. Red blood cells were lysed and leukocytes were fixed by adding FACS Lysing Solution (BD Bioscience, San Jose, CA, USA) following the manufacturer’s instructions. A minimum of 2 × 10^5^ PBMCs were acquired according to the forward light scatter versus side light scatter profile, and doublet discrimination was performed. The gating strategy is reported in Figure S4. Samples were processed on FACS Canto II system (Becton Dickinson, San Jose, CA, USA).

The purified cell viability assay was performed using Pacific Blue or APC Annexin V/SYTOX AADvanced Apoptosis Kit, for flow cytometry (Thermo Fisher Scientific, Waltham, MA, USA), following the manufacturer’s instructions. Briefly, this assay identifies the early apoptotic cells as annexin V^+^/SYTOX AADvanced^-^ and late apoptotic/necrotic cells as annexin V^+^/SYTOX AADvanced^+^. Samples were processed on MACS Quant Citofluorimeter (Miltenyi-Biotec). Results were analyzed by FlowJo X software (Tree Star Inc, Ashland, Wilmington, NC, USA).

For the evaluation of IFN-α and CXCL10 intracellular production, PBMCs and purified pDCs, stimulated as described above, were surface labelled with anti-BDCA-2 and anti-CD123 fluorochrome-conjugated antibodies. Subsequently, cells were fixed and permeabilized using the Inside Stain Kit (Miltenyi Biotec, Bergisch Gladbach, Germany) and the intracellular cytokine labelling was performed using anti IFN-α and anti-CXCL10/IP-10 fluorochrome-conjugated antibodies (panel #2 reported in Table S5). Samples were processed on MACS Quant Citofluorimeter (Miltenyi Biotec, Bergisch Gladbach, Germany). Results were analysed by FlowJo X software (Tree Star Inc, Wilmington, NC, USA).

### 4.6. Statistical Analysis

Patient characteristics were described at therapy initiation (T0). Categorical variables were reported as absolute frequencies and percentages and were compared across groups using the Fisher’s exact test. Continuous variables were expressed as median and interquartile range (IQR). The Wilcoxon–Mann–Whitney test and the Kruskal–Wallis test were used to compare variable distributions across two and more than two subgroups of patients, respectively. The association between the peripheral blood immune cell frequencies and tumor burden at T0 among MM patients was evaluated using the Spearman’s rank correlation coefficient. Peripheral blood leukocyte populations were measured at 1 month (T1) and at 4 months (T2) after therapy initiation (T0) in the group of MM patients that were BRAF-mutated. Changes in the peripheral blood leukocyte frequencies at 1 month (T1–T0) and at 4 months (T2–T0) were tested using the Wilcoxon signed-rank test. All patients were followed up after T0. Two endpoints (disease progression and death) were used to calculate the PFS and OS probability, respectively. PFS was defined as the time interval between T0 and the date of identification of progressive disease; OS was defined as the time interval between T0 and the date of death. Survival curves were calculated using Kaplan–Meier method, and differences in survival between subgroups of patients were tested using the log-rank test. For continuous variables, subgroups were defined using the median value as cut-off. Univariate Cox proportional hazard model were fitted to evaluate the role of the peripheral blood leukocyte populations and other established prognostic factors on the considered outcomes. Multivariable regression models were used to adjust the estimates for molecular profile and stage of the disease. The hazard ratios (HR), 95% confidence intervals (CI), and *p*-values from a Wald test were reported. The two-sample paired sign test and two-sample paired or unpaired Student’s *t*-test were used to compare groups from in vitro experiments. A two-tailed *p*-value < 0.05 was considered statistically significant.

The statistical analysis was performed using STATA 15 (StataCorp. 2017. Stata Statistical Software: Release 15. College Station, TX: StataCorp LLC.) or GraphPad Prism Software version 5 (GraphPad Software, San Diego, CA, USA).

## 5. Conclusions

In conclusion, pDCs from MM patients are severely impaired in their frequency and function. Findings emerged here suggest a relevant role for melanoma metabolism promoting lactic acidosis. In BRAF^V600+^ MM, short-term treatment is not associated to a full pDC recovery; however, TLR-9 agonists as adjuvant remain a valid therapeutic strategy for a proficient pDC activation. Monitoring the pDC compartment and functions might represent a clinically relevant tool for the selection of MM cases, which likely benefit from TLR-9 agonists as a completion of their treatment plan.

## Figures and Tables

**Table 1:** Clinical and molecular features of the study cohort at baseline (T0; *N* = 29).

**Clinical/Molecular Features**	**BRAF^V600+^ MM (*N* = 16)**			**NRAS^Q61+^ MM (*N* = 7)**			**BRAF^wt^/NRAS^wt^ (*N* = 6)**			
	***N***	***n***	**%**	***N***	***n***	**%**	***N***	***n***	**%**	***p***
Gender (males)	16	12	75	7	5	71.4	6	5	83.3	1
Stage	16			7			6			0.5
M1a		3	18.7		1	14.3		1	16.7	
M1b		5	31.3		0	0		2	33.3	
M1c		8	50		6	85.7		3	50	
Brain metastases	16	3	18.7	7	2	28.6	6	2	33.3	0.6
Tumor sites (≥3)	16	6	37.5	7	3	42.9	6	4	66.7	0.5
LDH (high *)	16	4	25	6	3	50	5	2	40	0.5
Therapy	16			7			6			
BRAFi		4	25							
BRAFi + MEKi		12	75							
Anti-CTLA-4					7	100		5	83.3	
Anti-PD-1								1	16.7	
	***N***	**Median** **(min-max)**	**SD**	***N***	**Median** **(min–max)**	**SD**	***N***	**Median** **(min–max)**	**SD**	***p***
Age	16	58.5 (23–76)	14.0	7	58.0 (48–76)	10.0	6	62.0 (53–79)	8.8	0.2
Tumor burden (mm)	15	116.5 (0–408.2)	127.6	7	77.9 (8–260.3)	89.0	6	136.4 (52.2–309.5)	98.0	0.8

* above the normal range; *p*: *p*-value; SD: standard deviation.

**Table 2:** Peripheral blood immune populations in healthy donors (HD; *N* = 25) and metastatic melanoma (MM) at baseline (T0; *N* = 29).

Immune Cell Population		MM (*N* = 29)			HD (*N* = 25)			
		*N*	Median	IQR	*N*	Median	IQR	*p* *
% pDCs on PBMCs		29	0.3	0.2–0.3	24	0.4	0.4–0.6	**0.0006**
% mDCs on PBMCs		29	0.3	0.2–0.5	15	0.5	0.4–0.7	**0.03**
% CD3^+^ on PBMCs		29	58.9	52.6–62.3	15	58	50.1–66.8	0.9
% CD4^+^ on PBMCs		29	28.7	20.1–39.0	15	34.6	27.4–40.8	0.1
**% IFN-α^+^ pDCs**	R848	27	48.5	30.2–65.0	25	71.5	64.3–74.7	**0.004**
	IMQ	27	21.1	8.7–41.7	24	38.3	29.4–45.1	**0.02**
	CpG	26	9.3	4.3–13.9	22	11.6	5–15.2	0.4
**% CXCL10^+^ pDCs**	R848	24	78.4	64.8–85.3	19	80.9	79.1–86.8	0.08
	IMQ	24	47.9	22.4–61.5	19	61.6	48.9–71.4	**0.03**
	CpG	25	7.1	3.7–17.1	20	10.2	2.9–14.8	0.7

IQR (interquartile range): Q1–Q3; * *p*: *p*-value. The significant *p*-values are highlighted in bold.

**Table 3:** Peripheral blood immune populations and lactate dehydrogenase (LDH) level in MM patients cohort at baseline (T0; *N* = 29).

Immune Cell Population		Normal LDH (*N* = 18)			High LDH * (*N* = 9)			
		*N*	Median	IQR	*N*	Median	IQR	*p*
*n*° leukocytes/µL		14	6805	5830–9270	9	9110	6860–12,050	0.08
% neutrophils on LK		14	54.7	47.2–68.5	8	66.2	54.9–74.3	0.1
% lymphocytes on LK		14	32.3	20.8–39	8	19.9	12.1–26.1	**0.02**
% monocytes on LK		14	8.3	7.5–11	8	7.5	6.1–10.3	0.2
% eosinophils on LK		14	2.1	1.6–2.5	8	0.8	0.1–3	0.1
% basophils on LK		14	0.6	0.4–0.8	8	0.5	0.2–0.6	0.3
% pDCs on PBMCs		18	0.3	0.2–0.4	9	0.1	0.1–0.2	**0.003**
% mDCs on PBMCs		18	0.4	0.3–0.5	9	0.2	0.1–0.2	**0.02**
% CD3^+^ on PBMCs		18	60.2	54.6–68.9	9	46.7	40.7–59.8	0.06
% CD4^+^ on PBMCs		18	30.9	25.5–42.6	9	23.8	17.8–27	**0.03**
**% IFN-α^+^ pDCs**	R848	17	56.7	35.1–82	9	43.0	25.9–58	0.2
	IMQ	17	27.7	14–56.4	9	13.6	5.4–27	0.08
	CpG	16	7.8	4.2–14.2	9	9.9	8.3–10.9	1
**% CXCL10^+^ pDCs**	R848	14	78.4	71.9–85.5	9	75.2	51.6–84.1	0.7
	IMQ	14	47.3	23.9–57.9	9	47.5	19–69	0.4
	CpG	15	5.2	2.9–11.7	9	17.1	5.7–36.8	0.1

IQR (interquartile range): Q1–Q3; *p*: *p*-value; LK: leukocytes; * referred to values above the normal range. The significant *p*-values are highlighted in bold.

**Table 4:** Correlation between tumor burden (mm) and peripheral blood immune cells of the MM patient cohort at baseline (T0; *N* = 29).

Immune Cell Population		*N*	Rho	*p*
*n*° leukocytes/µL		22	0.41	0.06
% neutrophils on LK		21	0.24	0.30
% lymphocytes on LK		21	−0.48	**0.03**
% monocytes on LK		21	−0.08	0.72
% eosinophils on LK		21	−0.22	0.34
% basophils on LK		21	−0.44	**0.05**
% pDCs on PBMCs		28	−0.51	**0.006**
% mDCs on PBMCs		28	−0.59	**0.001**
% CD3^+^ on PBMCs		28	−0.39	**0.04**
% CD4^+^ on PBMCs		28	−0.45	**0.02**
**% IFN-α^+^ pDCs**	R848	26	−0.36	0.07
	IMQ	26	−0.34	0.09
	CpG	25	−0.10	0.62
**% CXCL10^+^ pDCs**	R848	23	−0.28	0.20
	IMQ	23	−0.11	0.63
	CpG	24	0.11	0.61

Rho: Pearson correlation coefficient; LK: leukocytes; The significant *p*-values are highlighted in bold.

**Table 5:** Univariate and multivariate Cox regression models for OS in MM patients at baseline (T0; *N* = 29).

Immune Cell Population		Univariate			Multivariate °		
		HR	*p*	95% CI	HR	*p*	95% CI
*n*° leukocytes/µL		1.06 **	0.27	0.96–1.18			
% neutrophils on LK		1.01	0.61	0.96–1.07			
% lymphocytes on LK		**0.92**	**0.01**	0.87–0.98	0.95	0.06	0.90–1.00
% monocytes on LK		1.04	0.73	0.82–1.33			
% eosinophils on LK		0.79	0.33	0.49–1.27			
% basophils on LK		0.85 *	0.14	0.02–1.75			
% pDCs on PBMCs		0.61 *	**0.01**	0.68–1.06	0.72	0.06	0.52–1.01
% mDCs on PBMCs		0.84 *	0.24	0.63–1.12			
% CD3^+^ on PBMCs		**0.95**	**0.03**	0.91–1.00	0.98	0.54	0.94–1.04
% CD4^+^ on PBMCs		**0.93**	**0.01**	0.88–0.98	0.97	0.30	0.90–1.03
**% IFN-α^+^ pDCs**	R848	**0.98**	**0.04**	0.96–1.00	0.98	0.19	0.96–1.01
	IMQ	0.98	0.07	0.95–1.00			
	CpG	0.98	0.44	0.93–1.03			
**% CXCL10^+^ pDCs**	R848	**0.98**	**0.03**	0.96–1.00	0.99	0.25	0.97–1.01
	IMQ	1.00	0.72	0.97–1.02			
	CpG	**1.04**	**0.01**	1.01–1.07	**1.05**	**0.008**	1.01–1.08

LK: leukocytes; * HR associated with a 0.1 unit increase; ** HR associated with a 1000 unit increase; ° adjusted for molecular profile (NRAS^Q61+^ vs. BRAF^V600+^ or BRAF^wt^/NRAS^wt^) and stage of the disease (M1c vs. M1a or M1b). *p*: *p*-value. The significant *p*-values and relative hazard ratio are highlighted in bold.

**Table 6:** Variation of the peripheral blood immune populations at 1 month (T1; *N* = 12) and 4 months (T2; *N* = 9) from therapy’s initiation compared to the baseline (T0; *N* = 16) in the group of BRAF^V600+^ MM patients.

Immune Cell Population		Variation T1–T0				Variation T2–T0			
		*N*	Mean	SD	*p*	*N*	Mean	SD	*p*
*n*° leukocytes/µL		13	−810	1891	0.2	6	−1161	1260	0.1
% neutrophils on LK		13	−3.2	10.8	0.3	6	−10.3	12.7	0.1
% lymphocytes on LK		13	3.6	8.9	0.1	6	11.0	12.2	**0.05**
% monocytes on LK		13	1.0	3.6	0.2	6	0.1	1.9	0.8
% eosinophils on LK		13	0.7	1.8	0.2	6	−0.7	1.6	0.4
% basophils on LK		13	0.4	0.5	0.07	6	0.0	0.2	0.6
% pDCs on PBMCs		16	−0.1	0.1	**0.01**	9	−0.1	0.1	0.3
% mDCs on PBMCs		16	0.0	0.2	1.0	9	0.0	0.2	1.0
% CD3^+^ on PBMCs		16	−1.4	10.2	0.6	9	−4.5	9.7	0.5
% CD4^+^ on PBMCs		16	−0.1	6.6	1.0	9	−0.5	20.6	0.4
**% IFN-α^+^ pDCs**	R848	14	0.3	37.1	1.0	7	19.1	17.7	**0.03**
	IMQ	14	−2.0	34.0	1.0	7	8.6	14.6	0.1
	CpG	12	3.6	7.6	0.2	5	−0.1	9.1	0.9
**% CXCL10^+^ pDCs**	R848	12	−9.2	31.9	0.2	5	−4.1	9.1	0.4
	IMQ	12	−4.9	32.4	0.5	5	8.9	19.5	0.2
	CpG	12	−1.6	15.3	0.8	5	1.9	1.5	0.08

LK: leukocytes; *p*: *p*-value. The significant *p*-values are highlighted in bold.

## Supplementary Materials

The following are available online at https://www.mdpi.com/2072-6694/12/8/2085/s1, Figure S1: Lactate, glucose concentrations, and pH levels measured on melanoma cell line supernatants (SN-mel), Figure S2: Lactosis did not affect the viability of pDCs and T cells, Figure S3: BRAF and MEK inhibitors (BRAFi; MEKi) did not affect pDC viability and function, Figure S4: Gating strategy for the identification of peripheral blood immune populations, Table S1: Clinical data of the metastatic melanoma (MM) cohort. Table S2: Peripheral blood immune populations in the MM molecular groups at baseline (T0; *N* = 29), Table S3: Analysis of the overall survival (OS) and the progression-free survival (PFS) among MM patients (*N* = 29) during the follow-up, Table S4: Univariate and multivariate Cox regression models for PFS in MM patients at baseline (T0; *N* = 29), Table S5: Antibodies used for flow cytometry.
