# Supplementary material for: Plasmacytoid Dendritic Cell Impairment in Metastatic Melanoma by Lactic Acidosis
Source: Cancers (Basel). 2020 Jul 28;12(8):2085. doi: 10.3390/cancers12082085 (PMC7463681; doi:10.3390/cancers12082085)
Supplement: Supplementary file 1 [file cancers-12-02085-s001.pdf]

Supplementary Materials: Plasmacytoid Dendritic Cell Impairment in Metastatic Melanoma by Lactic Acidosis

Matilde Monti, Raffaella Vescovi, Francesca Consoli, Davide Farina, Daniele Moratto, Alfredo Berruti, Claudia Specchia and William Vermi

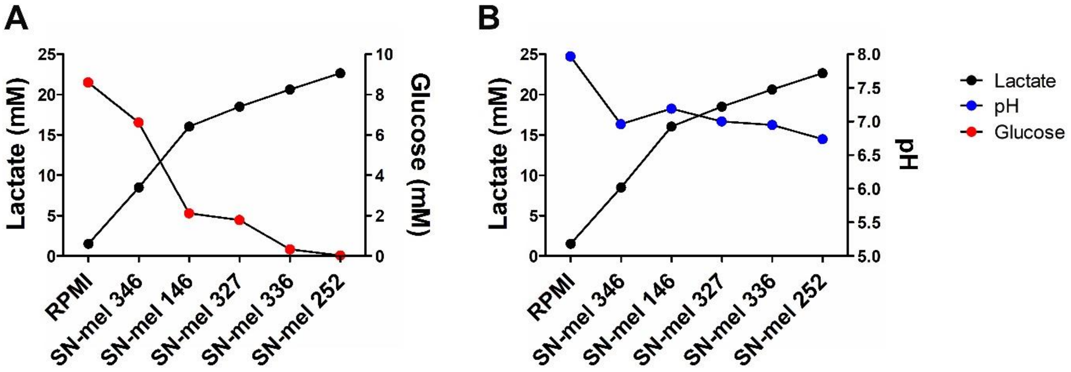

Figure S1. Lactate, glucose concentrations and pH levels measured on melanoma cell lines supernatants (SN–mel). Graphs show the concentration of lactate (black dots), glucose (red dots) (A) and the pH level (blue dots) **(B)** among different SN–mel and RPMI 1640 medium.

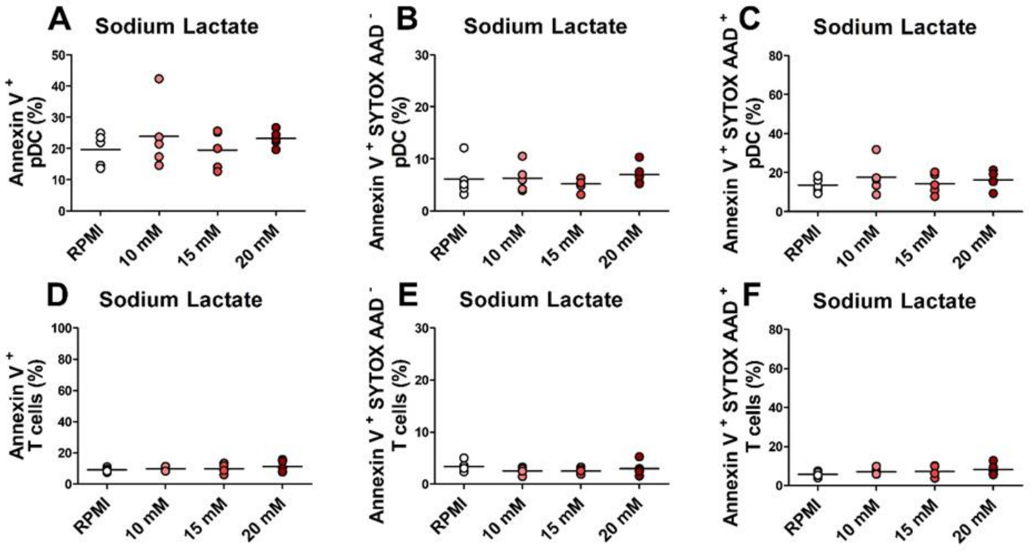

Figure S2. Lactosis did not affect the viability of pDCs and T cells. pDCs and T cells purified from buffy coats of HD were cultured in RPMI 1640 medium supplemented with 10% FBS containing Sodium Lactate (10 mM; 15 mM; 20 mM) (*n* = 5, **A–F**) for 24 hours. 20 ng/ml IL–3 was added to pDCs culture. The cellular viability was analyzed by Annexin V/SYTOX AADvanced staining in flow cytometry. Aligned dot plot graphs show the percentages of dead (**A, D**), early apoptotic (**B, E**) and late apoptotic or necrotic cells (**C, F**). Bars represent the mean of biological replicates. The statistical significance was calculated by two–sample paired sign test.

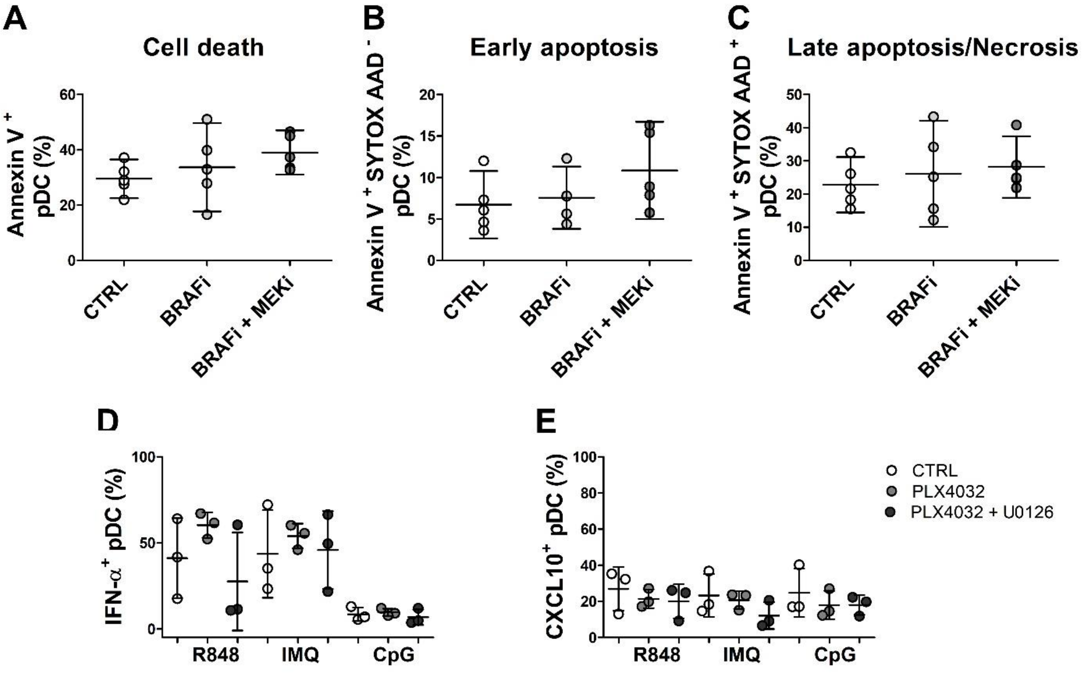

Figure S3. BRAF and MEK inhibitors (BRAFi; MEKi) did not affect pDC viability and function. pDCs purified from buffy coats of HD were cultured in RPMI 1640 medium supplemented with 10% FBS and IL–3 (CTRL) and treated with BRAFi (PLX4032 1 μM) alone or in combination with MEKi (U0126 12.5 μM). pDC viability was analyzed by Annexin V/SYTOX AADvanced staining in flow cytometry (n = 5) **(A–C)**. Scatter dot plot graphs illustrate the percentage of dead **(A)**, early apoptotic **(B)** or late apoptotic/necrotic cells **(C)**; bars represent the mean with 95% CI **(A–C).** pDCs were stimulated with R848 and Imiquimod (IMQ) for 2 hours **(D)** and 6 hours **(E)** and with CpG–ODN 2216 for 6 hours **(D, E)**. IFN–α **(D)** and CXCL10 **(E)** were analyzed by intracellular flow cytometry staining (n = 3). Scatter dot plot graphs show the percentage of positive pDCs evaluated on BDCA–2^{+}/CD123^{+} cells; bars represent the mean with SD **(D, E)**. The statistical significance was calculated by two–sample paired sign test.

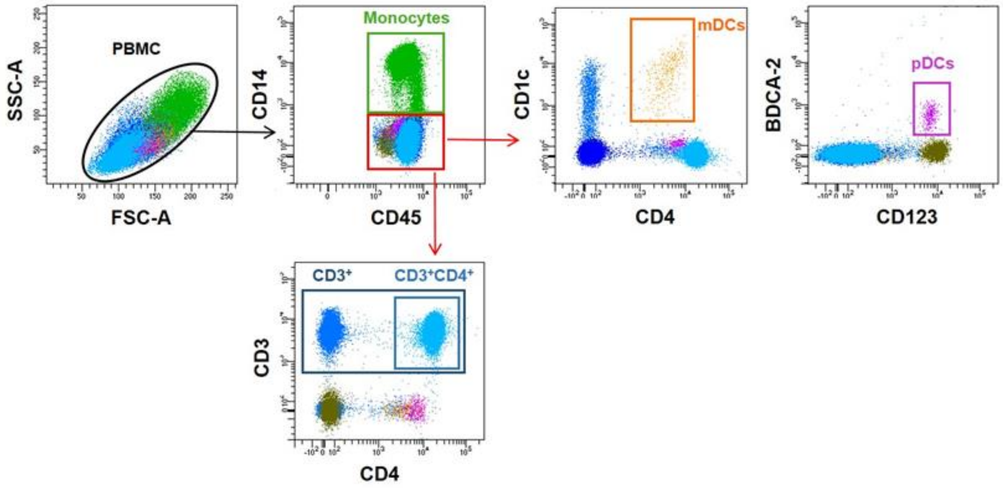

Figure S4. Gating strategy for the identification of peripheral blood immune populations. Flow cytometry analysis was performed on whole blood samples. The panels illustrate the gating strategy used to identify pDCs, mDCs, CD3^{+} and CD4^{+} T cells. At least 2x10^{5} PBMCs were acquired according to the forward light scatter versus side light scatter profile and doublet discrimination was performed. Among CD14^{-} cells, mDCs were recognized as CD45^{dim}/ CD1c^{+}/ CD4^{dim} cells, whereas pDCs were BDCA–2^{+}/CD123^{+}. T lymphocytes were recognized as CD45^{+}/CD3^{+}/CD4^{+}.

Table S1. Clinical data of the Metastatic Melanoma (MM) cohort.

Patient	Gender	Age	AJCC Staging	NRAS	BRAF	LDH (IU/L)	Brain metastasis	Tumor sites	Tumor Burden	RECIST	Therapy
#1	F	63	M1c	Q61R	wt	NA	No	>3	72.0	PD	Ipilimumab
#2	F	53	M1c	wt	wt	155 *	No	>3	52.2	PD	Ipilimumab
#3	M	45	M1c	wt	V600E	230 *	No	<3	174.4	PR	Vemurafenib
#4	F	50	M1b	wt	V600E	154 *	No	<3	43.1	PR	Vemurafenib
#5	M	43	M1c	wt	V600E	318 *	No	>3	408.2	PR	Dabrafenib + Trametinib
#6	M	63	M1a	wt	V600K	152 *	No	<3	35.0	SD	Vemurafenib
#7	M	49	M1c	wt	V600E	433 **	No	>3	227.9	PR	Dabrafenib + Trametinib
#8	M	67	M1c	Q61R	wt	286 *	No	<3	260.3	PD	Ipilimumab
#9	M	63	M1b	wt	V600E	230 *	No	<3	81.0	PR	Dabrafenib + Trametinib
#10	M	76	M1c	Q61K	wt	220 *	Yes	>3	137.1	PD	Ipilimumab
#11	M	64	M1c	wt	wt	558 *	Yes	>3	309.5	PD	Ipilimumab
#12	M	32	M1c	wt	V600E	160 *	No	>3	100.7	PR	Dabrafenib + Trametinib
#13	F	58	M1c	Q61K	wt	188 *	No	<3	8.0	PD	Ipilimumab
#14	M	50	M1b	Q61R	wt	247 *	No	<3	66.1	PD	Ipilimumab
#15	M	60	M1a	wt	wt	170 *	No	<3	83.3	PR	Ipilimumab
#16	M	67	M1c	wt	wt	320 *	Yes	>3	230.9	PD	Ipilimumab
#17	M	54	M1a	Q61R	wt	214 *	No	<3	77.9	PD	Ipilimumab
#18	M	48	M1c	Q61R	wt	1037 *	Yes	>3	209.5	PD	Ipilimumab
#19	M	62	M1c	wt	V600K	279 *	Yes	>3	389.9	PR	Dabrafenib + Trametinib
#20	M	79	M1b	wt	wt	181 *	No	>3	168.3	SD	Ipilimumab
#21	F	60	M1b	wt	V600E	227 *	No	<3	44.9	PR	Vemurafenib + Cobimetinib
#22	M	66	M1b	wt	V600K	199 *	No	<3	NA	PD	Vemurafenib + Cobimetinib
#23	M	57	M1c	wt	V600E	1910 *	No	>3	165.1	SD	Dabrafenib + Trametinib
#24	M	23	M1c	wt	V600E	526 *	Yes	>3	293.6	PD	Dabrafenib + Trametinib
#25	F	43	M1a	wt	V600E	143 *	No	<3	0.0	SD	Dabrafenib + Trametinib
#26	M	61	M1b	wt	V600E	188 *	No	<3	56.3	SD	Dabrafenib + Trametinib

Patient	Gender	Age	AJCC Staging	NRAS	BRAF	LDH (IU/L)	Brain metastasis	Tumor sites	Tumor Burden	RECIST	Therapy
#27	M	61	M1b	wt	wt	NA	No	<3	104.5	PR	Pembrolizumab
#28	M	77	M1a	wt	V600E	121 *	No	<3	184.9	CR	Dabrafenib + Trametinib
#29	F	69	M1c	wt	V600K	184 *	Yes	<3	116.5	PD	Dabrafenib

NA = Not Assessable; LDH reference range: * (134 – 236 IU/L), ** (313 – 618 IU/L); CR = Complete Response; PR = Partial Response; SD = Stable Disease; PD = Progressive Disease.

Table S2. Peripheral blood immune populations in the MM molecular groups at baseline (T0; *N* = 29).

Immune Cell Population		MM with $BRAF^{V600+}$ (*N* = 16)			MM with $NRAS^{Q61+}$ (*N* = 7)			MM $BRAF^{wt}/NRAS^{wt}$ (*N* = 6)			
		N	Median	IQR	*N*	Median	IQR	*N*	Median	IQR	*p* *
n° leukocytes/µL		14	7350	6610–9660	5	8290	4930–9110	4	8040	6635–9315	0.9
% neutrophils on LK		14	62.5	54.1–69	4	46.1	36.3–59.3	4	51.5	47.5–64.8	0.3
% lymphocytes on LK		14	24.6	17–30.4	4	31.4	11.7–41.1	4	34.5	25.2–37.4	0.4
% monocytes on LK		14	8.3	7.1–10.5	4	7	3.5–9.8	4	9.4	7.1–11.7	0.6
% eosinophils on LK		14	2.1	0.5–2.5	4	1.4	0.7–2	4	1.7	1.1–3.9	0.6
% basophils on LK		14	0.5	0.4–0.5	4	0.8	0.4–1	4	0.6	0.6–0.8	0.1
% pDCs on PBMCs		16	0.3	0.2–0.3	7	0.2	0.1–0.5	6	0.2	0–0.3	0.6
% mDCs on PBMCs		16	0.3	0.2–0.5	7	0.4	0.1–0.7	6	0.3	0.2–0.3	0.8
% $CD3^+$ on PBMCs		16	59.4	52.6–61.6	7	53.1	40.7–68.9	6	61.3	57.2–66.8	0.4
% $CD4^+$ on PBMCs		16	28.7	24.5–40.8	7	17.8	16.8–40.1	6	30.9	28.7–34.4	0.2
**% IFN–α^+ pDCs**	R848	16	47.7	28.1–72.2	7	60.4	10.3–65	4	58.6	49.6–72.2	0.7
	IMQ	16	24.4	9.9–49	7	27	8.7–38.2	4	13.3	8.7–41.4	0.9
	CpG	14	9.8	4.1–14.5	7	9.3	6.3–10.6	5	9.3	5.4–11.7	0.9
**% $CXCL10^+$ pDCs**	R848	13	81.8	71.9–85.8	7	80	4.8–85.2	4	74.5	62.7–76.1	0.3
	IMQ	13	48.8	46.4–61.8	7	45.7	14.7–61.2	4	34.8	21.4–55.3	0.5
	CpG	13	8.8	3.7–12.3	7	5.7	4.6–23.7	5	7.1	2.9–7.7	0.9

IQR (interquartile range): Q1–Q3; LK: leukocytes; * p: p–value referred to comparison among the three different molecular groups of MM patients.

Table S3. Analysis of the overall survival (OS) and the progression free survival (PFS) among MM patients (*N* = 29) during the follow–up.

		**MM (*N* = 29)**	**BRAF^{V600+} MM (*N* = 16)**	**NRAS^{Q61+} MM (*N* = 7)**	**BRAF^{wt}/NRAS^{wt} MM (*N* = 6)**
**OS**	number (%) of deaths	18 (62)	10 (63)	6 (86)	2 (33)
	median time (months)	14	17	5	not reached
	survival at 12 months* (95% CI)	0.52 (0.33–0.68)	0.62 (0.35–0.81)	0.14 (0.01–0.46)	0.67 (0.19–0.90)
**PFS**	number (%) of progressions**	22 (76)	10 (63)	7 (100)	5 (83)
	median time (months)	4	12	3	3
	survival at 12 months* (95% CI)	0.30 (0.15–0.47)	0.50 (0.25–0.71)	–	–

*months from therapy initiation; **all deaths went into disease progression.

Table S4. Univariate and multivariate Cox regression models for PFS in MM patients at baseline (T0; *N* = 29).

**Immune Cell Population**		**Univariate**			**Multivariate°**		
		**HR**	***p***	**95% CI**	**HR**	***p***	**95% CI**
**n° leukocytes/μL**		1.04**	0.51	0.93–1.15			
% neutrophils on LK		0.99	0.80	0.95–1.04			
% lymphocytes on LK		0.96	0.14	0.91–1.01			
% monocytes on LK		1.07	0.57	0.86–1.32			
% eosinophils on LK		0.75	0.18	0.49–1.14			
% basophils on LK		0.97*	0.76	0.79–1.19			
% pDCs on PBMCs		0.76*	0.08	0.56–1.04			
% mDCs on PBMCs		0.91*	0.45	0.03–4.42			
% $CD3^+$ on PBMCs		0.97	0.14	0.93–1.01			
% $CD4^+$ on PBMCs		**0.95**	**0.02**	0.91–0.99	0.98	0.6	0.94–1.04
**% IFN–α^+ pDCs**	R848	0.99	0.31	0.97–1.01			
	IMQ	0.99	0.26	0.97–1.01			
	CpG	0.97	0.24	0.92–1.02			
**% $CXCL10^+$ pDCs**	R848	0.98	0.08	0.97–1.00			
	IMQ	0.99	0.61	0.97–1.02			
	CpG	1.02	0.24	0.99–1.04			

LK: leukocytes; *HR associated with a 0.1 unit increase; **HR associated with a 1000 unit increase; p: p–value; ºadjusted for molecular profile (NRAS^{Q61+} vs BRAF^{V600+} or BRAF^{wt}/NRAS^{wt}) and stage of the disease (M1c vs M1a or M1b). The significant *p*–values and relative hazard ratio are highlighted in bold.

Table S5. Antibodies used for flow cytometry.

**Reagent**	**Clone**	**Conjugation**	**Source**
**Panel #1**			
CD303	AC144	FITC	Miltenyi Biotec
CD123	AC145	VioBlue	Miltenyi Biotec
CD16	3G8	PE	Becton Dickson
CD8	HIT8a	PerCP Cy5.5	Becton Dickson
CD3	UCHT1	PE Cy7.7	Becton Dickson
CD45RA	2D1	APC H7	Becton Dickson
CD4	RPA–T4	V450	Becton Dickson
CD1c	F10/21A3	PE	Becton Dickson
CD19	HIB19	FITC	Becton Dickson
CD14	M5E2	FITC	Becton Dickson
**Panel #2**			
CD303	AC144	FITC	Miltenyi Biotec
CD123	AC145	VioBlue	Miltenyi Biotec
IFN–α	REA1013	APC	Miltenyi Biotec
CXCL10/IP–10	J034D6	PE	Biolegend

© 2020 by the authors. Submitted for possible open access publication under the terms and conditions of the Creative Commons Attribution (CC BY) license (http://creativecommons.org/licenses/by/4.0/).
